# A Bioactivated *Lepidium latifolium* Formulation Disrupts Mitochondrial Bioenergetics and Metabolic Adaptation in KRAS-Mutant Cancer Cells

**DOI:** 10.3390/molecules31162779

**Published:** 2026-08-10

**Authors:** María Conde-Rioll, Aiora Cenigaonandia-Campillo, Silvia Sanz, José Antonio Esteban, Oscar Aguilera

**Affiliations:** 1 Department of Research, Development and Innovation (R&D&I), Soria Natural S.A., 42162 Garray, Soria, Spain; celcult@sorianatural.es (M.C.-R.); celcult2@sorianatural.es (S.S.); jaesteban@sorianatural.es (J.A.E.); 2 Translational Oncology Division, OncoHealth Institute, IIS-Fundación Jiménez Díaz-UAM, 28040 Madrid, Madrid, Spain; aioraceni@gmail.com; 3 Department of Food Technology and Nutrition, Universidad Católica de Murcia (UCAM), Campus de los Jerónimos 135, 30107 Guadalupe, Murcia, Spain

**Keywords:** *Lepidium latifolium*, glucosinolates, KRAS, pancreatic cancer, colorectal cancer, metabolism, CETP, oxidative stress

## Abstract

Pancreatic ductal adenocarcinoma (PDAC) and colorectal cancer (CRC) are aggressive malignancies frequently driven by oncogenic Kirsten rat sarcoma viral oncogene homolog (KRAS) mutations associated with metabolic reprogramming and resistance to apoptosis. In this study, we evaluated the antitumor and anti-inflammatory activity of a *Lepidium latifolium* L.-derived formulation (CTP) enriched in glucosinolate hydrolysis products in KRAS-mutant colorectal and pancreatic cancer models. The formulation was designed to promote the generation of the epithionitrile 1-cyano-2,3-epithiopropane (CETP) through iron-dependent myrosinase-mediated sinigrin hydrolysis. CTP induced dose-dependent cytotoxicity and morphological alterations consistent with apoptosis in KRAS-mutant cancer cell lines. Treatment significantly reduced mitochondrial membrane potential, ATP production, oxygen consumption rate (OCR), and extracellular acidification rate (ECAR), indicating severe bioenergetic impairment. In parallel, CTP downregulated the metabolic and proliferative regulators C-myc, PKM2, GLUT1, and Cyclin E1. RNA-seq analysis revealed extensive transcriptional reprogramming associated with oxidative stress, metabolic adaptation, and cell-cycle regulation. In addition, CTP significantly suppressed nitric oxide, IL-6, and IL-8 production in LPS-stimulated RAW 264.7 macrophages. These findings demonstrate that glucosinolate-derived metabolites from *L. latifolium* interfere with metabolic and inflammatory pathways critical for KRAS-driven tumor survival and support the therapeutic potential of *Brassicaceae*-derived epithionitriles as multitarget anticancer agents.

## 1. Introduction

Although cancer encompasses more than 200 distinct diseases, its global incidence and mortality continue to rise substantially worldwide. According to recent epidemiological estimates, the number of newly diagnosed cancer cases is projected to increase from 18.1 million in 2018 to approximately 29.5 million by 2040 [1]. Among these malignancies, pancreatic ductal adenocarcinoma (PDAC) and colorectal cancer (CRC) represent major public health challenges because of their elevated mortality, metastatic potential, and resistance to current therapeutic approaches. PDAC remains one of the deadliest malignancies, whereas CRC is among the most frequently diagnosed cancers worldwide [1,2].

Pancreatic ductal adenocarcinoma is characterized by extensive desmoplastic stroma, profound metabolic adaptation, and intrinsic chemoresistance. Activating mutations in KRAS are detected in more than 90% of PDAC cases and constitute central molecular drivers of tumor initiation, progression, invasion, and metabolic rewiring [3].

Similarly, KRAS mutations occur in approximately 40–45% of colorectal cancers and are associated with poor prognosis and reduced therapeutic response [4]. Constitutive KRAS activation stimulates downstream signaling pathways including RAF-MEK-ERK and PI3K-AKT-mTOR, thereby promoting proliferation, survival, angiogenesis, and resistance to apoptosis [5]. In addition to its role in oncogenic signaling, KRAS profoundly reprograms tumor metabolism. KRAS-driven tumors exhibit enhanced glycolytic flux, increased glucose uptake mediated by GLUT1 overexpression, elevated lactate production, and increased dependence on glutamine utilization, lipid biosynthesis, and mitochondrial plasticity to sustain proliferation under nutrient-limited conditions [6,7]. Furthermore, KRAS-mutant tumors display increased oxidative stress and altered mitochondrial homeostasis, resulting in elevated reactive oxygen species (ROS) production and selective susceptibility to compounds capable of disrupting redox balance and mitochondrial integrity [8]. These metabolic liabilities have stimulated growing interest in natural products capable of simultaneously targeting signaling and metabolic pathways involved in tumor progression. Dietary and environmental factors are known to significantly influence cancer risk and progression. In this context, epidemiological studies have consistently associated diets rich in cruciferous vegetables with reduced incidence of several malignancies, particularly colorectal and gastrointestinal cancers [9]. Cruciferous vegetables belonging to the *Brassicaceae* family contain high concentrations of glucosinolates, sulfur-containing phytochemicals that, upon enzymatic hydrolysis, generate biologically active metabolites including isothiocyanates, nitriles, thiocyanates, and epithionitriles [10]. Several *Brassicaceae* species, including broccoli (*Brassica oleracea*), mustard (*Brassica juncea*), and watercress (*Nasturtium officinale*), have been extensively investigated because their glucosinolate-derived isothiocyanates, particularly sulforaphane and allyl isothiocyanate, exhibit well-established chemopreventive and anticancer activities. However, comparatively less attention has been paid to sinigrin-derived epithionitriles and their potential biological properties.

Considerable evidence suggests that these hydrolysis products, rather than intact glucosinolates, are primarily responsible for the chemopreventive and antitumor activities associated with *Brassicaceae* species.

**Figure 1:**
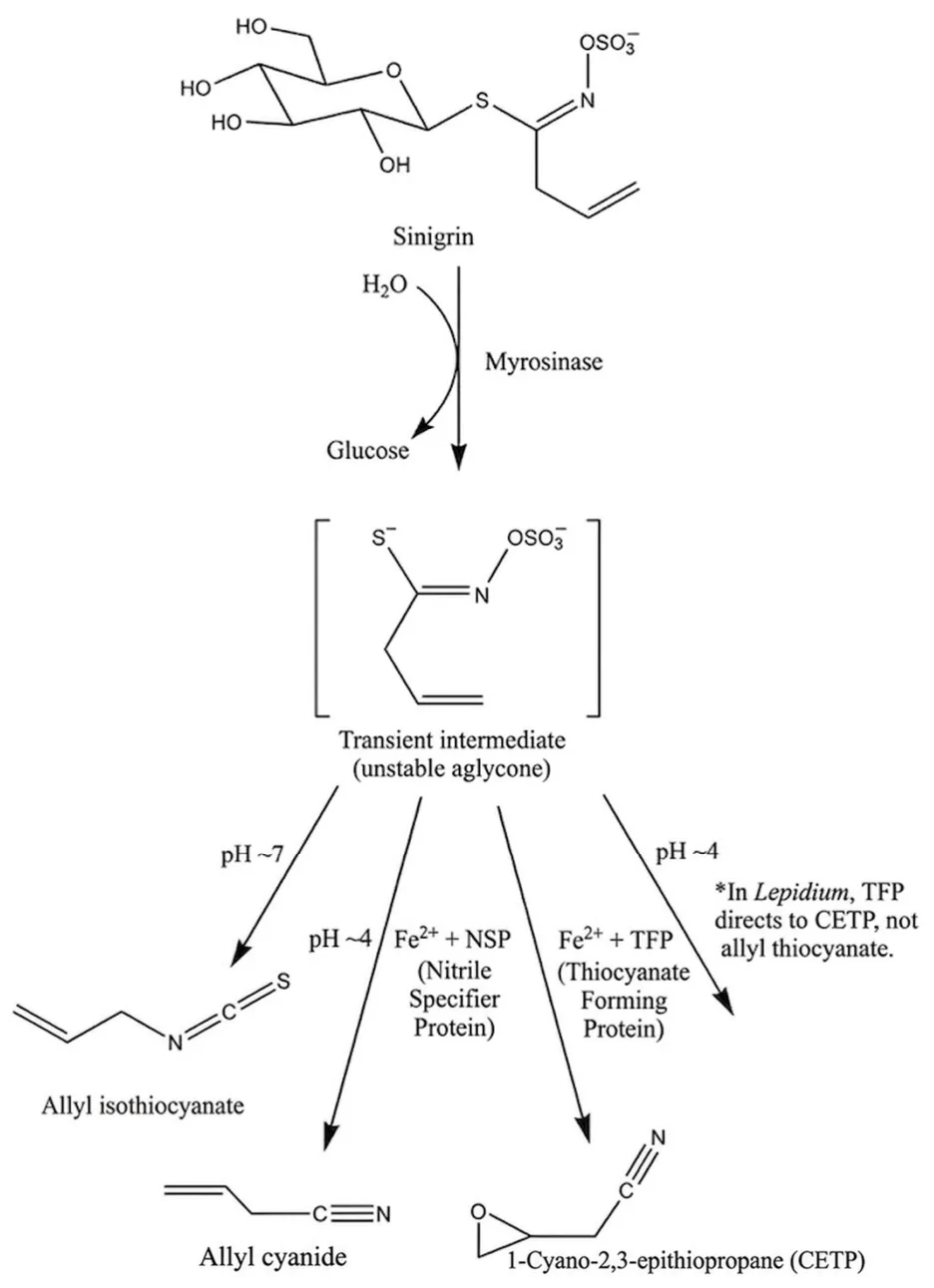
Myrosinase-catalyzed hydrolysis of the glucosinolate sinigrin and formation of distinct degradation products under different reaction conditions. Enzymatic cleavage of the thioglucosidic bond generates a transient, *cis*-configurated (*Z*-isomer) aglycone intermediate. Under neutral conditions (pH ~7), this intermediate spontaneously rearranges into allyl isothiocyanate. Under acidic conditions (pH ~4), the reaction is directed toward allyl cyanide, a process facilitated by the Fe^2+^-dependent nitrile specifier protein (NSP). In the presence of the Fe^2+^-dependent thiocyanate-forming protein (TFP), the pathway is redirected; in *Lepidium*, TFP specifically promotes the formation of the epithionitrile 1-cyano-2,3-epithiopropane (CETP) instead of allyl thiocyanate. Glucose release upon initial hydrolysis is also indicated.

*Lepidium latifolium* L., a member of the *Brassicaceae* family traditionally used in folk medicine, represents a particularly rich natural source of the glucosinolate sinigrin. Extracts from this species have demonstrated antioxidant, anti-inflammatory, antimicrobial, detoxifying, and antitumor properties [11]. Upon tissue disruption, endogenous myrosinase catalyzes the hydrolysis of sinigrin, producing an unstable aglycone intermediate capable of generating distinct degradation products depending on cellular pH, iron availability, and the activity of the epithiospecifier protein (ESP) and the thiocyanate-forming protein (TFP) [10]. Figure 1 illustrates the enzymatic degradation pathway of sinigrin and the formation of allyl isothiocyanate, allyl cyanide, allyl thiocyanate, and the epithionitrile 1-cyano-2,3-epithiopropane (CETP). Among the different hydrolysis products derived from sinigrin, CETP has been identified as one of the major ones generated during hydrolysis mediated by ESP and/or TFP in the presence of ferrous ions as a cofactor, and has been proposed as a principal contributor to the antitumor activity of *Lepidium latifolium*-derived preparations [12].

Previous studies demonstrated that both synthetic and plant-derived CETP exhibit potent proapoptotic activity in multiple tumor cell lines. Flow cytometry and biochemical analyses revealed that CETP-induced apoptosis involves mitochondrial dysfunction, generation of reactive oxygen species, loss of mitochondrial transmembrane potential, and activation of caspase-dependent signaling pathways [12,13]. These findings have subsequently been supported and extended by independent studies investigating the biological activity of epithionitriles. Kelleher et al. demonstrated that CETP activates cytoprotective genes and phase II detoxification enzymes, thereby enhancing cellular resistance to electrophilic and oxidative stress, suggesting that this epithionitrile modulates redox homeostasis in addition to its direct cytotoxic effects [14]. Likewise, Hanschen et al. reported that CETP induced cell death associated with mitochondrial dysfunction, oxidative stress, caspase activation, and both apoptotic and necrotic features in hepatocellular carcinoma cells, while also observing comparable effects in primary cells [13].

Together, these studies establish CETP as a biologically active glucosinolate-derived metabolite with context-dependent effects on oxidative stress, mitochondrial function, and cell survival.

Importantly, CETP-mediated apoptosis was significantly attenuated by overexpression of antiapoptotic proteins such as Bcl-2 and Bcl-xL, as well as by inhibition of Fas/CD95 signaling and caspase-8 activation, indicating the involvement of both intrinsic and extrinsic apoptotic pathways [12]. The ability of CETP to modulate mitochondrial integrity and oxidative stress is particularly relevant in KRAS-driven malignancies, which exhibit increased dependence on redox adaptation and metabolic plasticity for tumor survival. In addition, extracts of *Lepidium latifolium* significantly inhibited tumor growth in HT-29 human colon cancer xenograft models, further supporting the therapeutic potential of this natural product [12].

The increasing recognition of cancer metabolism as a therapeutic target has stimulated interest in natural products capable of interfering with glycolysis, mitochondrial bioenergetics, inflammatory signaling, and lipid metabolism [4,15,16]. In this context, glucosinolate-rich plants and their epithionitrile derivatives represent attractive candidates for the development of innovative anticancer strategies because of their ability to simultaneously modulate multiple hallmarks of cancer. Based on these considerations, a tablet formulation containing dried *L. latifolium* leaf extract together with selected excipients, including ferrous sulfate, was developed to promote in situ hydrolysis within the gastrointestinal tract and favor the formation of CETP while minimizing the instability associated with this volatile epithionitrile compound. The incorporation of ferrous ions was specifically intended to enhance epithionitrile generation during enzymatic hydrolysis and thereby increase the production of biologically active metabolites after oral administration.

The main objective of the present study was to evaluate the antitumor and anti-inflammatory properties of the CETP formulation through in vitro experimental approaches and to identify molecular and metabolic pathways associated with its activity in KRAS-mutant pancreatic and colorectal cancer models. Particular emphasis was placed on the modulation of mitochondrial dysfunction, oxidative stress, inflammatory mediators, and KRAS-associated signaling pathways involved in tumor survival, proliferation, and metabolic adaptation.

Although glucosinolate-derived isothiocyanates have been extensively investigated as anticancer compounds, much less is known about sinigrin-derived epithionitriles. Unlike previous studies based on purified glucosinolates or isothiocyanates, the present work evaluates a bioactivated *Lepidium latifolium* formulation specifically designed to promote in situ epithionitrile generation and investigates its effects on KRAS-mutant colorectal and pancreatic cancer metabolism through integrated metabolic, transcriptomic and inflammatory analyses.

## 2. Results

### 2.1. Chemical Characterization of the CTP Formulation

CTP is a bioactivated glucosinolate-based formulation obtained from young leaves of *Lepidium latifolium* L. (*Brassicaceae*) and specifically designed to promote the generation of biologically active sulfur-containing metabolites following enzymatic hydrolysis. Unlike conventional botanical extracts, the formulation incorporates both glucosinolate substrates and the endogenous enzymatic machinery required for their biotransformation, thereby being designed to promote the in situ generation of reactive epithionitrile metabolites with potential biological activity. Soria Natural developed this formulation, which was patented in 2014 under the title “Detoxifying composition of alkenylglucosinolate” (No. ES2376716).

**Figure 2:**
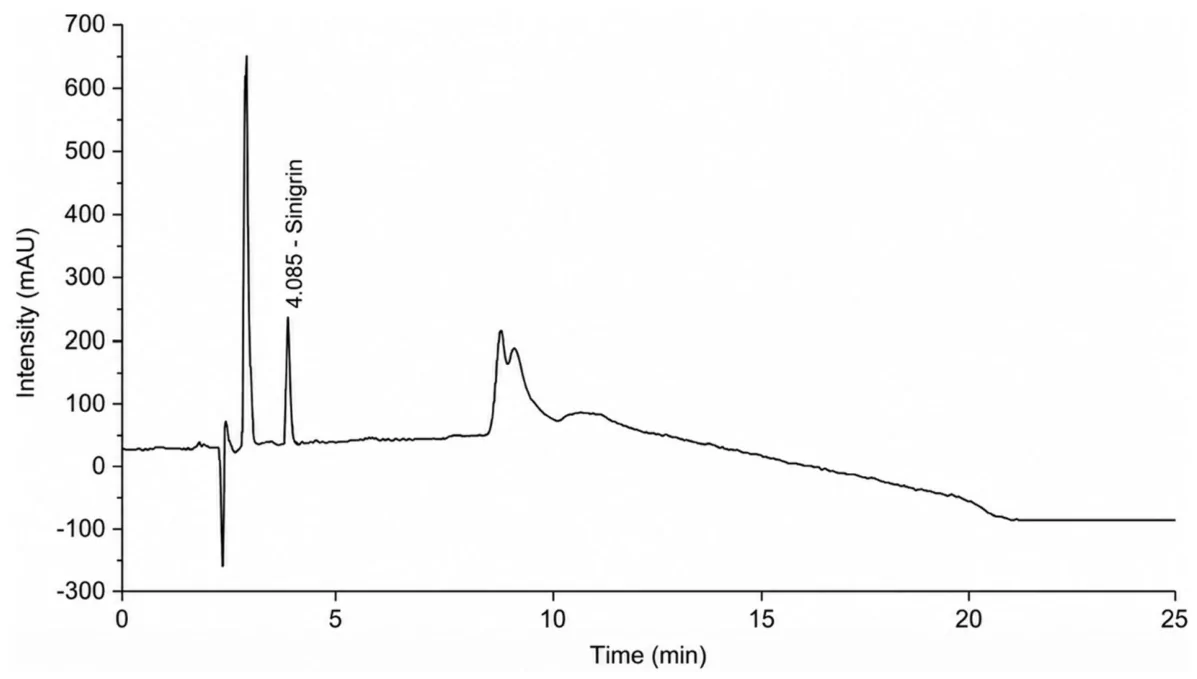
Quantification of sinigrin in the CTP formulation by high-performance liquid chromatography coupled with diode array detection (HPLC-DAD). The major chromatographic peak is observed at a retention time (tR) of 4.085 min, corresponding to a concentration of 33.52 mg of sinigrin per gram of CTP preparation.

To confirm the presence and quantify the major glucosinolate present in the formulation, sinigrin content was analyzed by high-performance liquid chromatography coupled with diode-array detection (HPLC-DAD). As shown in Figure 2, the chromatogram exhibited a major peak at a retention time (tR) of 4.085 min, corresponding to a sinigrin concentration of 33.52 mg g^−1^ in the CTP preparation. These results confirm the presence of sinigrin as the principal glucosinolate substrate available for subsequent enzymatic bioactivation within the formulation.

To further characterize the phytochemical composition of the CTP formulation beyond its glucosinolate content, an LC-Orbitrap high-resolution mass spectrometry analysis was performed. As shown in Table 1, the formulation contains several flavonoids, including rutin, hyperoside/isoquercitrin, luteolin-7-*O*-glucuronide, apigenin-7-*O*-glucoside, diosmin, luteolin, quercetin, and kaempferol, among others. The total flavonoid content was 12.8 mg/100 mL.

Although these flavonoids may contribute to the antioxidant and anti-inflammatory properties of the formulation, the present study primarily focuses on the glucosinolate-derived sulfur-containing metabolites generated after sinigrin hydrolysis, which constitute the mechanistic basis of CTP bioactivation.

**Figure 3:**
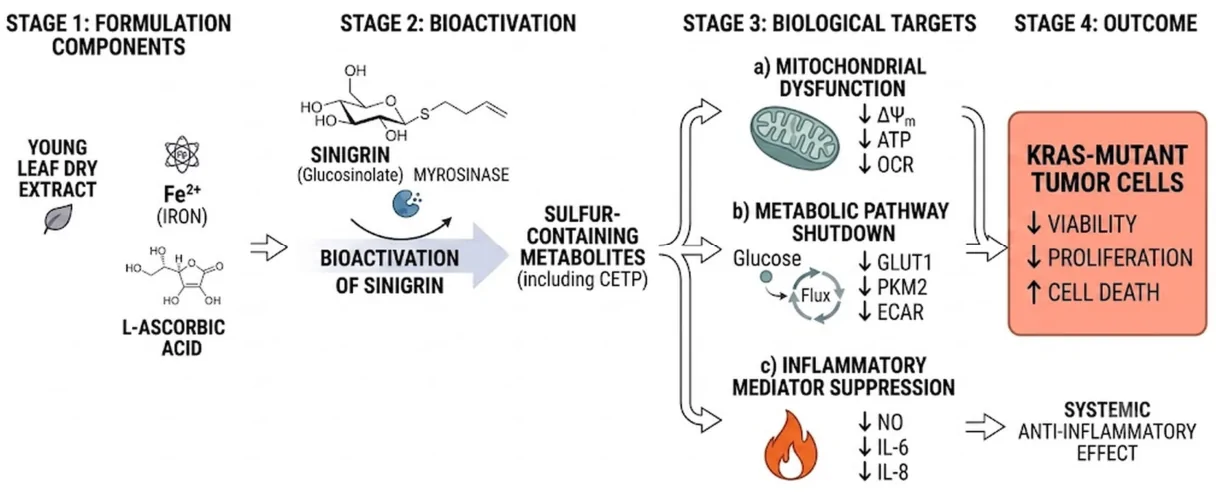
Proposed therapeutic mechanism of the Lepidium latifolium-derived CTP formulation in KRAS-mutant tumors. The schematic illustrates the sequential stages underlying the proposed mode of action of the Lepidium latifolium-derived CTP formulation. In Stage 1, the formulation comprises young leaf dry extract, Fe^2+^, and L-ascorbic acid. In Stage 2, sinigrin is enzymatically hydrolyzed by myrosinase, generating sulfur-containing metabolites, including 1-cyano-2,3-epithiopropane (CETP), depending on the activity of endogenous specifier proteins such as thiocyanate-forming protein (TFP) and epithiospecifier protein (ESP). In Stage 3, these metabolites induce mitochondrial dysfunction (decreased mitochondrial membrane potential [ΔΨm], ATP production, and oxygen consumption rate [OCR]), suppress glycolytic metabolism (reduced GLUT1 and PKM2 expression and extracellular acidification rate [ECAR]), and attenuate inflammatory signaling (reduced nitric oxide [NO], IL-6, and IL-8 production). These coordinated effects lead to reduced viability and proliferation and increased cell death in KRAS-mutant tumor cells, while contributing to a systemic anti-inflammatory effect (Stage 4). The diagram was designed with the assistance of the AI tool ChatGPT (OpenAI, GPT-5.5).

As shown in Figure 3, each tablet contains powdered young leaves of *L. latifolium* (304.64 mg), dry extract of young leaves of *L. latifolium* (205.70 mg), ascorbic acid, ferrous sulfate, and standardized amounts of sinigrin (30 mg), endogenous myrosinase activity (5 UI), and epithiospecifier protein (ESP; 200 μg). The coordinated presence of these components is mechanistically relevant because myrosinase catalyzes glucosinolate hydrolysis, whereas ESP and ferrous ions redirect the reaction toward epithionitrile formation rather than isothiocyanate generation.

The proposed bioactivation pathway of sinigrin within the CTP formulation is illustrated in Figure 3. Myrosinase-catalyzed hydrolysis generates an unstable aglycone intermediate that, depending on reaction conditions, may produce different sulfur-containing metabolites. The proposed bioactivation pathway of sinigrin within the CTP formulation is illustrated in Figure 3. Myrosinase-catalyzed hydrolysis generates an unstable aglycone intermediate that may give rise to different sulfur-containing metabolites depending on the activity of endogenous specifier proteins and the reaction conditions. In the presence of endogenous specifier proteins, such as thiocyanate-forming protein (TFP) or related specifier proteins, together with Fe^2+^ as an essential cofactor, sinigrin hydrolysis can be redirected toward the formation of epithionitriles, including 1-cyano-2,3-epithiopropane (CETP), rather than the corresponding isothiocyanate. In contrast, Fe^2+^ alone is insufficient to promote CETP formation and instead favors the generation of simple nitriles.

Collectively, these findings indicate that CTP constitutes a chemically and functionally defined glucosinolate-activating formulation enriched in reactive sulfur-containing metabolites capable of modulating oxidative stress, mitochondrial function, inflammatory signaling, and cancer-associated metabolic pathways. As summarized in Figure 3, these metabolites may contribute to mitochondrial dysfunction, oxidative stress imbalance, metabolic reprogramming, and induction of apoptotic signaling pathways in KRAS-mutant pancreatic and colorectal cancer models.

### 2.2. Effects of Lepidium latifolium Leaf Preparation (CTP) on KRAS-Mutant Colon and Pancreatic Human Cancer Cell Lines

**Figure 4:**
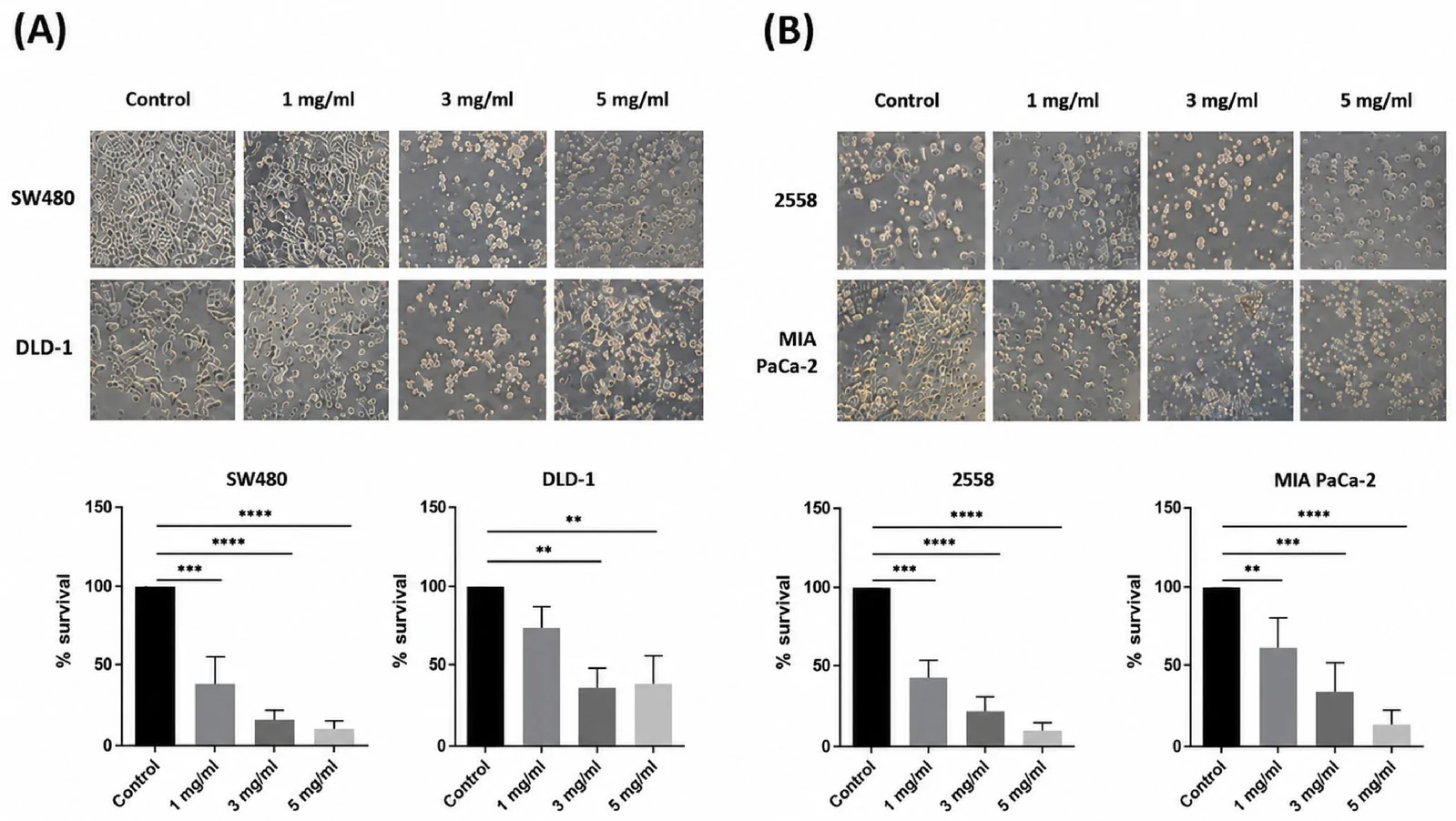
Effects of the tested extract on the viability and morphology of colorectal and pancreatic cancer cell lines. Representative phase-contrast micrographs and quantitative analysis of cell survival after treatment with increasing concentrations of the extract (1, 3, and 5 mg/mL). (**A**) Human colorectal cancer cell lines SW480 and DLD-1. (**B**) Human pancreatic cancer cell lines PL45 and MIA PaCa-2. Cell viability was normalized to untreated control cells (100%) and expressed as percentage survival. Data are presented as mean ± SD from at least three independent experiments. Statistical significance was determined relative to the control group using one-way ANOVA followed by multiple comparison tests. ** *p* < 0.01, *** *p* < 0.001, **** *p* < 0.0001.

Treatment with the *Lepidium latifolium* leaf preparation (CTP) induced marked morphological alterations and significantly reduced cell viability in KRAS-mutant colorectal (SW480 and DLD-1) and pancreatic (MIA PaCa-2 and PL45) cancer cell lines in a concentration-dependent manner (Figure 4).

Under control conditions, all cell lines displayed the typical morphology of adherent epithelial cells forming dense monolayers.

Treatment with 1 mg/mL CTP produced a moderate decrease in cell density, accompanied by early morphological alterations, including partial cell rounding and detachment. These changes became more evident at 3 mg/mL, where cells exhibited extensive rounding, loss of adherence, and increased cellular debris. At (5 mg/mL) a marked reduction in the number of adherent cells and a predominance of rounded floating cells were observed.

Quantitative analysis confirmed a significant concentration-dependent reduction in cell viability (Figure 4). In SW480 cells, viability decreased to approximately 40–50% at 1 mg/mL, 15–20% at 3 mg/mL, and below 15% at 5 mg/mL relative to untreated controls.

DLD-1 cells exhibited lower sensitivity, with viability values of approximately 75–80% at 1 mg/mL and 35–45% at 3–5 mg/mL. Similar concentration-dependent reductions in viability were observed in the pancreatic cancer cell lines MIA PaCa-2 and PL45, although PL45 cells appeared slightly more sensitive at the highest concentration tested.

Overall, these results demonstrate that CTP treatment induces marked morphological alterations accompanied by a significant concentration-dependent reduction in cell viability in KRAS-mutant colorectal and pancreatic cancer cell lines.

### 2.3. Modulation of Metabolic and Cell-Cycle Regulatory Proteins by CTP in KRAS-Mutant Colorectal and Pancreatic Cancer Cells

**Figure 5:**
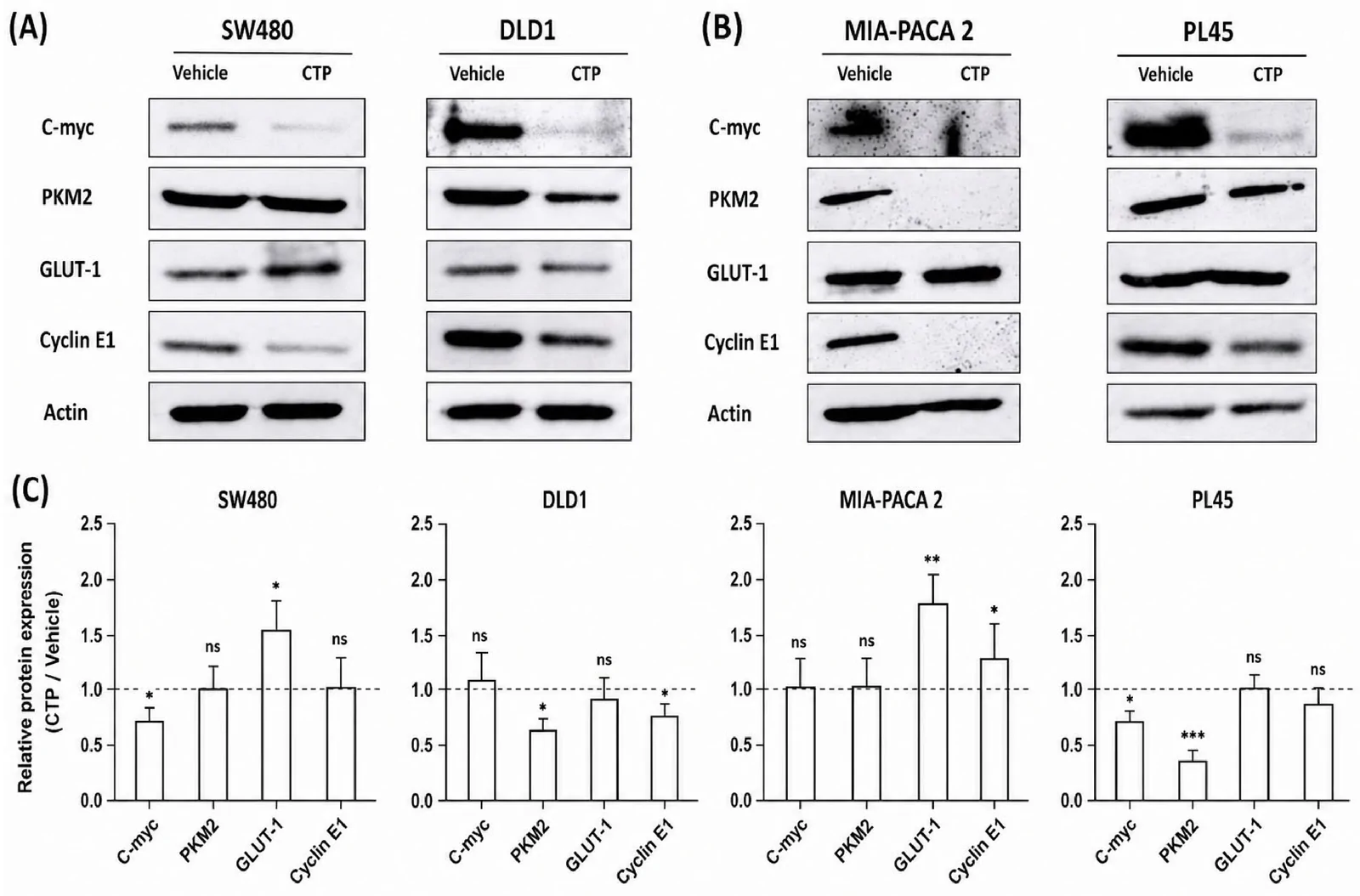
Effect of CTP treatment on the expression of metabolic and cell-cycle regulatory proteins in KRAS-mutant colorectal and pancreatic cancer cell lines. KRAS-mutant colorectal cancer cell lines SW480 and DLD1 (**A**), and pancreatic ductal adenocarcinoma cell lines MIA PaCa-2 and PL45 (**B**), were treated with CTP (1 mg/mL) for 24 h. Protein expression levels of C-myc, PKM2, GLUT1, and Cyclin E1 were evaluated by Western blot analysis. β-Actin was used as a loading control to confirm equal protein loading. Representative immunoblots from at least three independent experiments are shown. (**C**) Densitometric analysis of relative protein expression levels normalized to β-Actin and expressed as CTP/Vehicle ratio. Data are presented as mean ± SD from three independent experiments. Statistical significance was determined relative to vehicle-treated controls (* *p* < 0.05, ** *p* < 0.01, *** *p* < 0.001; ns, not significant).

Treatment of two KRAS-mutant colorectal cancer cell lines (SW480 and DLD-1) and two pancreatic ductal adenocarcinoma (PDAC) cell lines (MIA PaCa-2 and PL45) with 1 mg/mL CTP produced a marked and reproducible downregulation of the key oncogenic regulators c-Myc, PKM2, Cyclin E1, and GLUT1. Consistent with these findings, Western blot analysis demonstrated reduced expression of several proteins involved in cellular metabolism and cell-cycle regulation compared with vehicle-treated controls (Figure 5A,B). β-Actin was used as a loading control to confirm equal protein loading.

CTP treatment modulated the expression of the metabolic and cell-cycle regulators c-Myc, PKM2, GLUT1, and Cyclin E1, although the magnitude and direction of the response varied among the different KRAS-mutant cell lines. The most consistent decreases were observed for c-Myc and Cyclin E1, whereas PKM2 and GLUT1 exhibited cell line-dependent changes. Densitometric analysis confirmed that the response was heterogeneous across the different models, with statistically significant reductions detected for specific proteins in selected cell lines (Figure 5C). The decrease was statistically significant for most proteins and cell lines analyzed, whereas some differences did not reach statistical significance.

Overall, these results show that CTP modulates the expression of proteins associated with metabolic regulation, glucose uptake, and cell-cycle progression in KRAS-mutant colorectal and pancreatic cancer cells.

### 2.4. RNA-Seq Analysis Reveals Transcriptional Reprogramming Induced by CTP in KRAS-Mutant Pancreatic Cancer Cells

To further investigate the molecular effects of CTP, transcriptomic profiling was performed in the KRAS-mutant pancreatic ductal adenocarcinoma cell line MIA PaCa-2 following treatment with CTP (5 mg/mL) for 24 h. Total RNA was extracted and analyzed by next-generation sequencing.

**Figure 6:**
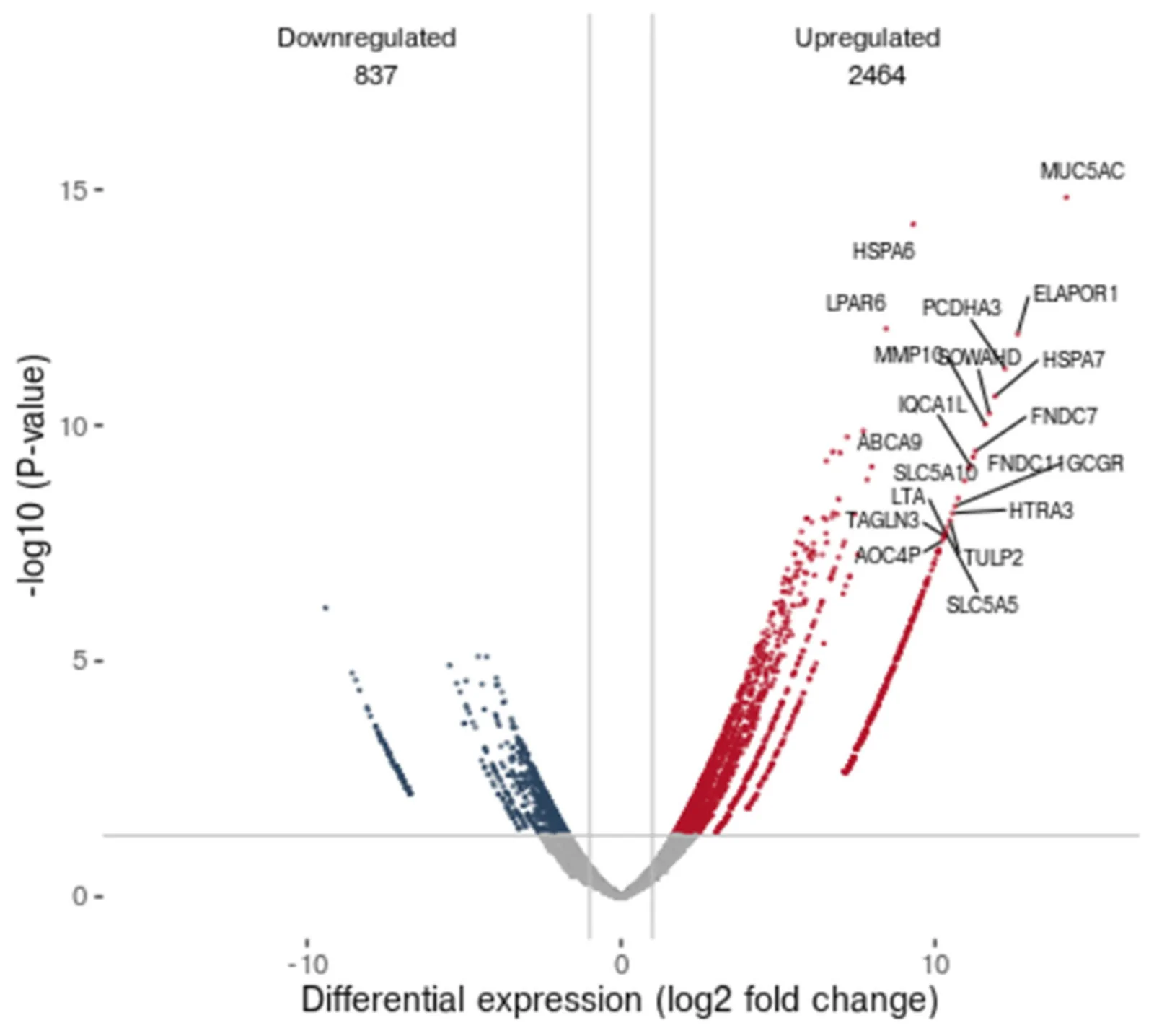
Transcriptomic alterations induced by CTP treatment in KRAS-mutant MIA PaCa-2 pancreatic cancer cells. Differential gene expression analysis was performed by RNA-seq in MIA PaCa-2 cells treated with CTP (5 mg/mL, 24 h) compared with untreated control cells. The volcano plot displays significantly upregulated genes (red) and downregulated genes (blue), plotted according to log2 fold change (x-axis) and −log10 (*p*-value) (y-axis). A total of 2464 genes were significantly upregulated, whereas 837 genes were downregulated following CTP treatment. Selected genes with high statistical significance and biological relevance, including *MUC5AC*, *HSPA6*, *PCDHA3*, *MMP10*, *SLC5A5*, *HTRA3*, and *TAGLN3*, are indicated. The transcriptomic profile demonstrates that CTP induces extensive transcriptional reprogramming associated with stress responses, metabolic adaptation, cell-cycle regulation, and tumor-associated signaling pathways in KRAS-mutant pancreatic cancer cells.

Differential expression analysis using edgeR identified extensive transcriptional changes in response to CTP treatment. As shown in Figure 6, a total of 2464 genes were significantly upregulated, whereas 837 genes were downregulated compared with untreated control cells. The volcano plot illustrates the distribution of differentially expressed genes according to log2 fold change and statistical significance.

The 19 most significantly differentially expressed genes are listed in Table 2, including the corresponding Ensembl identifiers, log2 fold changes, *p*-values, and false discovery rates (FDRs). Among the significantly regulated transcripts were *MUC5AC*, *HSPA6*, *HSPA7*, *MMP10*, *FNDC7*, *HTRA3*, *CYP4F11*, *SLC5A6*, *CDKN1A*, and *CDC25A*. Several Ensembl identifiers could not be assigned to annotated gene symbols and may correspond to uncharacterized transcripts or pseudogenes.

Overall, RNA-seq analysis demonstrated that CTP treatment induced widespread alterations in gene expression in MIA PaCa-2 cells.

### 2.5. CTP Induces Bioenergetic Collapse in KRAS-Mutant Colon and Pancreatic Cancer Cells Through Dual Inhibition of Glycolysis and Mitochondrial Respiration

**Figure 7:**
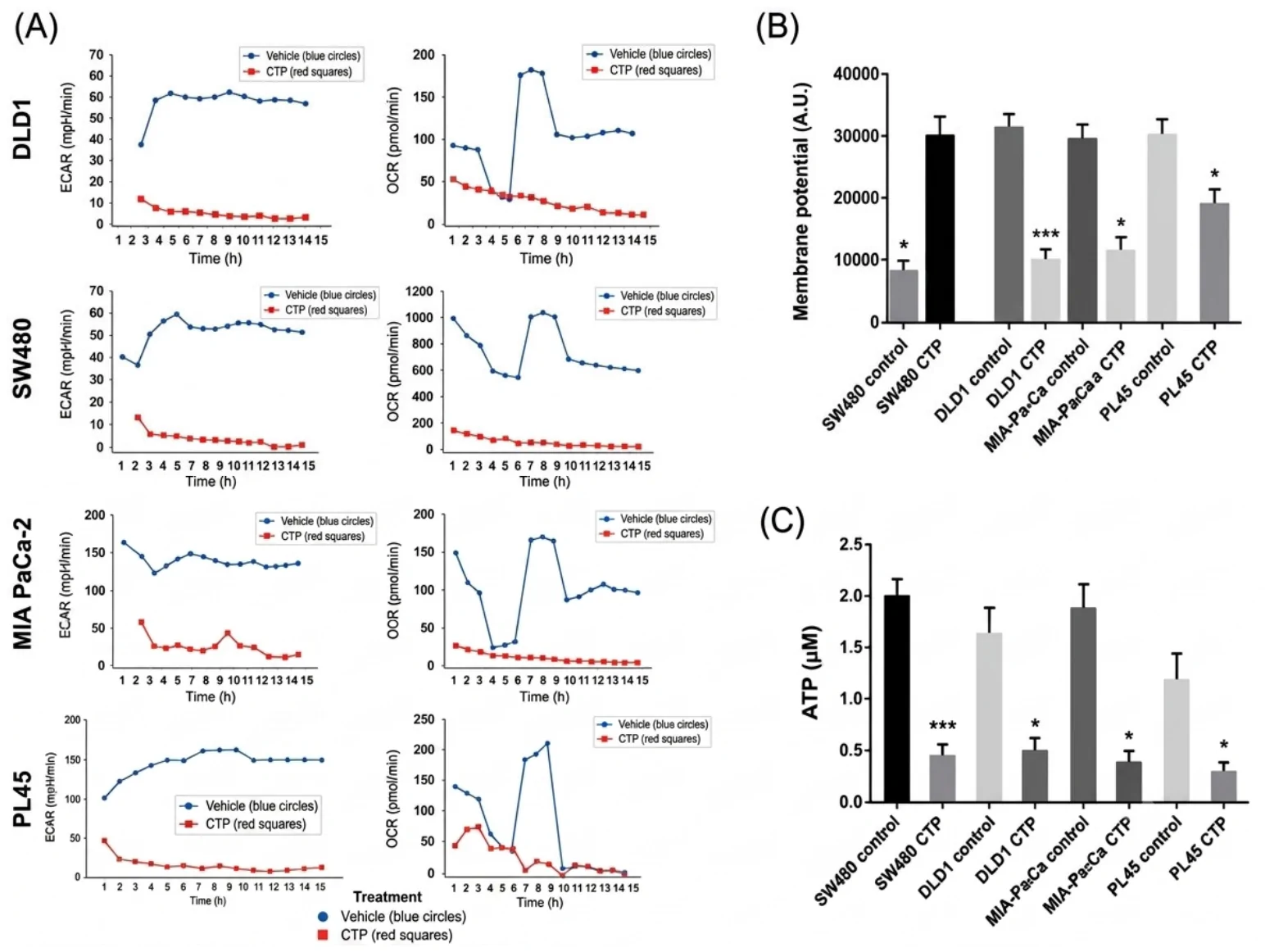
Effect of CTP on cellular bioenergetics in KRAS-mutant colorectal and pancreatic cancer cells. The impact of CTP on metabolic function was evaluated in two KRAS-mutant colorectal cancer cell lines (SW480 and DLD1) and two KRAS-mutant pancreatic ductal adenocarcinoma cell lines (MIA PaCa-2 and PL45). Cells were treated with vehicle or 1 mg/mL CTP under identical experimental conditions. (**A**) Real-time extracellular flux analysis showing extracellular acidification rate (ECAR; left panels), a surrogate of glycolytic activity, and oxygen consumption rate (OCR; right panels), a measure of mitochondrial respiration. Traces represent kinetic measurements following sequential injections of metabolic modulators (as indicated), and values are expressed as mpH/min for ECAR and pmol O_2_/min for OCR. (**B**) Quantification of mitochondrial membrane potential (ΔΨm), expressed in arbitrary units (A.U.), relative to control cells. (**C**) Intracellular ATP levels (µM) measured under the same conditions. Data represent mean ± SD of three independent experiments and are normalized to cell number/protein content where applicable. Statistical significance was determined using one-way ANOVA followed by Tukey’s post hoc test, with * *p* < 0.05 and *** *p* < 0.001 versus corresponding control.

To evaluate the effects of CTP on cellular metabolism, extracellular flux analyses were performed in KRAS-mutant colorectal (SW480 and DLD1) and pancreatic (MIA PaCa-2 and PL45) cancer cell lines following treatment with CTP (1 mg/mL). As shown in Figure 7A, CTP treatment markedly reduced both oxygen consumption rate (OCR) and extracellular acidification rate (ECAR) in all tested cell lines compared with untreated controls, indicating suppression of mitochondrial respiration and glycolytic activity. In addition to the reduction in OCR and ECAR, CTP treatment altered mitochondrial membrane potential (ΔΨm) (Figure 7B). A significant decrease was observed in DLD-1, MIA PaCa-2, and PL45 cells, whereas SW480 cells did not exhibit the same pattern and showed a slightly higher ΔΨm than control under the experimental conditions. Nevertheless, intracellular ATP levels were significantly reduced in all cell lines (Figure 7C) indicating an overall impairment of cellular bioenergetics.

Overall, CTP markedly impaired cellular bioenergetics in KRAS-mutant colorectal and pancreatic cancer cells, as evidenced by consistent reductions in OCR, ECAR, and ATP levels. Although the effects on mitochondrial membrane potential differed among cell lines, with SW480 cells not showing the same decrease observed in the other models, the overall findings support a substantial disruption of cellular energy metabolism following CTP treatment.

These findings are consistent with the dependence of KRAS-driven tumors on mitochondrial metabolism and bioenergetic adaptation mechanisms [17,18,19]. Notably, pancreatic cancer cell lines showed greater sensitivity to CTP-induced bioenergetic disruption, consistent with the reported dependence of pancreatic ductal adenocarcinoma cells on mitochondrial metabolism and noncanonical redox pathways [18,20]. Collectively, these findings indicate that CTP disrupts bioenergetic homeostasis in KRAS-mutant colorectal and pancreatic cancer cells, although the magnitude of the response varies among cell lines.

### 2.6. CTP Suppresses LPS-Induced Inflammatory Mediators in RAW 264.7 Macrophages

**Figure 8:**
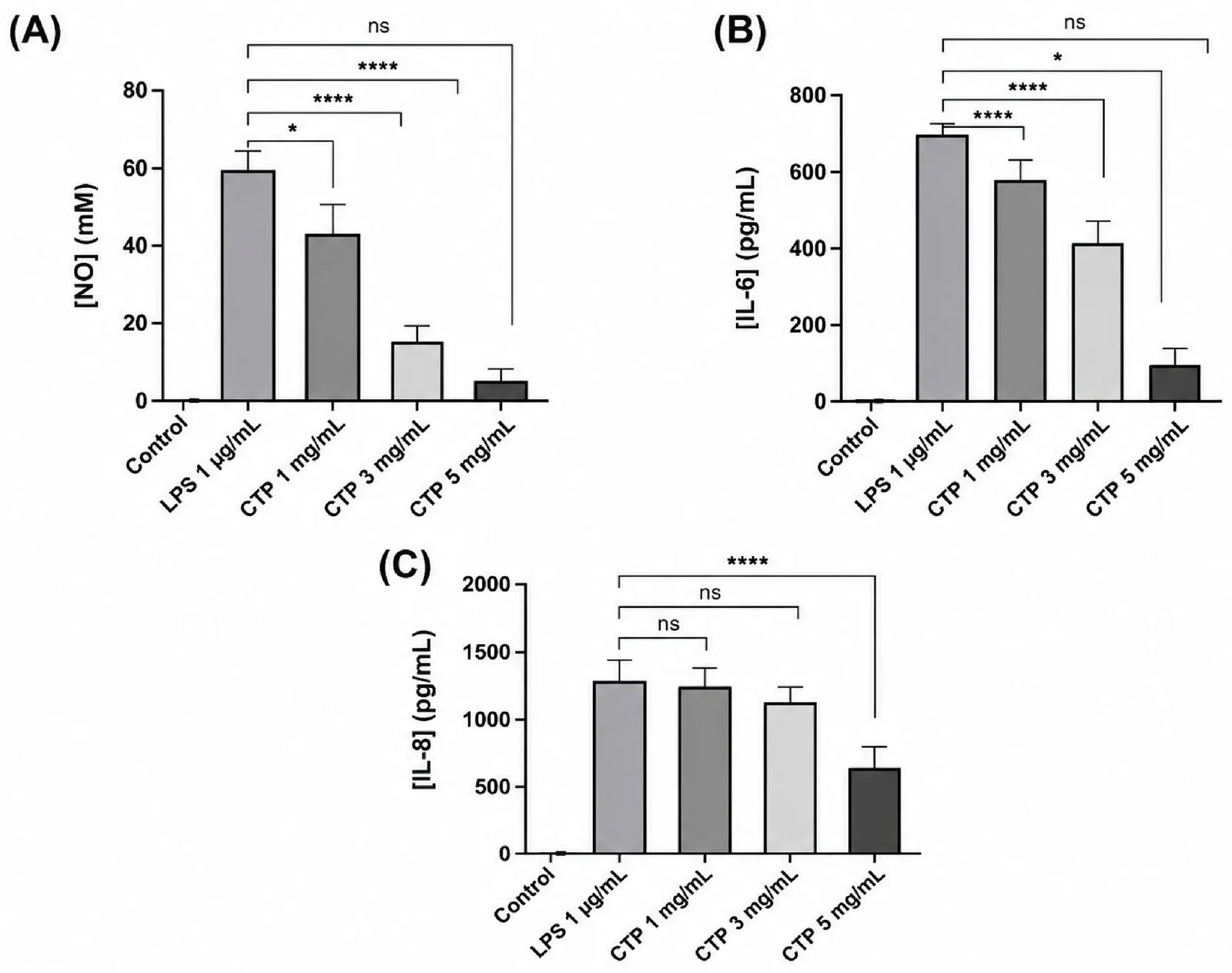
Anti-inflammatory effect of CTP on LPS-stimulated RAW 264.7 murine macrophages. Cells were stimulated with LPS and treated with CTP at different concentrations (1, 3, and 5 mg/mL). (**A**) Nitrite accumulation (Griess assay), used as an indirect measure of nitric oxide (NO) production, (**B**) IL-6 secretion, and (**C**) IL-8 secretion. LPS stimulation markedly increased the production of inflammatory mediators compared with untreated control cells, whereas CTP treatment significantly reduced NO, IL-6, and IL-8 levels in a concentration-dependent manner, with the strongest inhibitory effect observed at 5 mg/mL. Data are presented as mean ± SD of three independent experiments. Statistical analysis was performed using one-way analysis of variance (ANOVA) followed by Tukey’s multiple comparison test. Statistical significance is indicated as *p* < 0.05 (*) and *p* <0.0001 (****); ns = not significant.

The anti-inflammatory activity of CTP was evaluated in LPS-stimulated RAW 264.7 murine macrophages, a widely used in vitro model for studying inflammatory signaling pathways and innate immune activation [21,22]. As shown in Figure 8, LPS stimulation markedly increased nitrite accumulation, as determined by the Griess assay, reflecting enhanced nitric oxide (NO) production compared with untreated control cells and confirming effective induction of the inflammatory response. Treatment with CTP significantly reduced nitrite accumulation in a concentration-dependent manner. While 1 mg/mL CTP produced a moderate reduction, concentrations of 3 and 5 mg/mL induced a more pronounced inhibitory effect, reducing nitrite levels close to basal values.

Similarly, CTP treatment decreased IL-6 secretion in a concentration-dependent manner following LPS stimulation. The strongest inhibitory effect was observed at 5 mg/mL, where cytokine levels were markedly reduced compared with LPS-treated controls. In addition, IL-8 production was also attenuated after CTP treatment, particularly at the highest concentration tested.

The coordinated reduction in nitrite accumulation (reflecting NO production), IL-6 secretion, and IL-8 secretion demonstrates that CTP exerts significant anti-inflammatory activity in activated macrophages. These findings are consistent with the reported involvement of nitric oxide and pro-inflammatory cytokines in macrophage-mediated inflammatory responses and oxidative stress-associated processes [23,24,25,26,27,28,29,30,31]. Similar anti-inflammatory effects have previously been described for several natural bioactive compounds capable of suppressing macrophage activation and inflammatory mediator production [32,33,34,35]. Collectively, these results indicate that CTP induces a marked concentration-dependent suppression of inflammatory mediator production in LPS-stimulated RAW 264.7 macrophages.

## 3. Discussion

The present study provides robust evidence that the CTP formulation, derived from *Lepidium latifolium*, exerts potent multitarget antitumor and anti-inflammatory activities in colorectal cancer (CRC) and pancreatic ductal adenocarcinoma (PDAC) cellular models driven by oncogenic KRAS. Our findings demonstrate that the therapeutic activity of this preparation relies on its ability to simultaneously disrupt metabolic adaptation, mitochondrial bioenergetics, proliferative signaling pathways, and the production of inflammatory mediators.

The mechanistic foundation of the CTP formulation represents a key aspect of its design.

HPLC-DAD analysis confirmed sinigrin as the predominant glucosinolate present in the formulation (Figure 2). Unlike conventional botanical preparations, CTP preserves both the glucosinolate substrate and the endogenous enzymatic machinery required for its bioactivation, including myrosinase and endogenous specifier proteins involved in glucosinolate hydrolysis, together with ferrous ions and ascorbic acid (Figure 3).

Under these conditions, iron-dependent hydrolysis of sinigrin is expected to favor the formation of epithionitriles rather than isothiocyanates. Previous studies in *Lepidium* species and related *Brassicaceae* have demonstrated that myrosinase-catalyzed hydrolysis of sinigrin in the presence of ESP and Fe^2+^ promotes the generation of epithionitriles, nitriles, and other reactive sulfur-containing metabolites.

Among these, 1-cyano-2,3-epithiopropane (CETP) has consistently been identified as one of the major epithionitrile products and has been associated with the antitumor activity of *L. latifolium*-derived preparations. Nevertheless, enzymatic hydrolysis also generates additional sulfur-containing metabolites, including allyl cyanide and allyl isothiocyanate, which may contribute to the biological activity of the formulation. Therefore, the antitumor and anti-inflammatory effects observed in the present study are likely to result from the combined activity of several glucosinolate-derived metabolites rather than from CETP alone.

The observed biological effects are therefore likely mediated by glucosinolate-derived sulfur-containing metabolites expected to be generated upon enzymatic bioactivation, among which CETP has previously been identified as a major epithionitrile and is proposed as one potential contributor.

Consistent with this proposed mechanism, CTP treatment induced a marked reduction in cell viability in both KRAS-mutant colorectal and pancreatic cancer cell lines, together with pronounced morphological alterations, including cell rounding, shrinkage, and loss of adhesion (Figure 4). These morphological features are consistent with previous reports describing apoptosis induced by CETP, a glucosinolate-derived epithionitrile, supporting the hypothesis that the CTP formulation, through its bioactivation products, interferes with cellular pathways required for the survival of KRAS-driven malignancies.

Although all cell lines responded to treatment, differences in sensitivity were observed, with SW480 cells being more susceptible than DLD-1 cells. This variability may reflect differences in metabolic plasticity or dependence on KRAS-regulated signaling pathways, although additional studies will be required to elucidate the molecular basis of these differences.

At the molecular level, these phenotypic changes were accompanied by the coordinated downregulation of C-myc, PKM2, GLUT1, and Cyclin E1 (Figure 5). These proteins constitute a central regulatory network controlling proliferation and metabolic adaptation in KRAS-driven tumors. C-myc is a master transcription factor downstream of KRAS signaling that regulates genes involved in biomass production, glycolysis, and cell growth. Among its downstream targets are PKM2, which promotes aerobic glycolysis and metabolic reprogramming, and GLUT1, the major glucose transporter responsible for sustaining the increased glucose uptake required by highly proliferative cancer cells. In parallel, Cyclin E1 promotes G1/S-phase transition and uncontrolled cell-cycle progression and is frequently overexpressed in colorectal and pancreatic cancers. Therefore, the coordinated reduction in the expression of these proteins suggests that CTP interferes with both the metabolic and proliferative programs required for the maintenance of KRAS-mutant tumor cells. The molecular alterations identified by Western blot were consistent with the functional metabolic impairment observed in the bioenergetic analyses.

This molecular disruption of metabolic pathways translated directly into a generalized, functional bioenergetic collapse, as evidenced by extracellular flux analyses (Seahorse). CTP treatment drastically blocked both the extracellular acidification rate (ECAR, an indicator of glycolytic flux) and the oxygen consumption rate (OCR, a reflection of mitochondrial respiration) across all cell lines (Figure 7A). This dual metabolic shutdown was accompanied by a severe loss of mitochondrial membrane potential (ΔΨ) (Figure 7B) and, consequently, a drastic and statistically significant depletion of intracellular ATP levels (Figure 7C). Pancreatic cancer cells mutated in KRAS, which are frequently characterized by a strict mitochondrial addiction and the utilization of non-canonical redox pathways, proved to be extremely susceptible to this CTP-induced energetic crisis (Figure 7B,C). This demonstrates that the alteration in cellular redox balance provoked by the formulation’s metabolites overwhelms the tumor’s buffering and adaptive capacity, forcing an unsustainable energetic collapse.

At first glance, the induction of oxidative stress in cancer cells may appear inconsistent with the anti-inflammatory activity observed in activated macrophages. However, these effects are not mutually exclusive and are likely to be highly cell type-dependent. Cancer cells typically maintain elevated basal levels of reactive oxygen species (ROS) and rely on adaptive antioxidant mechanisms to preserve redox homeostasis. Further disruption of this balance can overwhelm their antioxidant capacity, promoting mitochondrial dysfunction and cell death. In contrast, in activated macrophages, glucosinolate-derived metabolites may attenuate inflammatory signaling pathways, thereby reducing the production of nitric oxide and pro-inflammatory cytokines. Thus, the pro-oxidant effects observed in tumor cells and the anti-inflammatory effects detected in macrophages likely reflect distinct context-dependent cellular responses rather than contradictory biological activities.

Although anticancer effects have been reported for several glucosinolate-rich *Brassicaceae* species, these studies have mainly focused on isothiocyanates such as sulforaphane and allyl isothiocyanate. Therefore, some of the biological effects observed here may also be shared by other glucosinolate-containing plants. However, the present study differs in that it investigates a chemically characterized *Lepidium latifolium* formulation specifically designed to promote in situ epithionitrile generation and provides an integrated mechanistic characterization in KRAS-mutant colorectal and pancreatic cancer models. These findings broaden the current understanding of glucosinolate-derived metabolites beyond the extensively studied isothiocyanates and support the therapeutic potential of epithionitrile-enriched formulations.

These findings broaden the current understanding of glucosinolate-derived metabolites beyond the extensively studied isothiocyanates and support the therapeutic potential of epithionitrile-enriched formulations. RNA-seq analysis further supported the broad molecular impact of CTP treatment by revealing extensive transcriptional remodeling in KRAS-mutant MIA PaCa-2 cells. Rather than affecting a limited number of signaling molecules, CTP induced widespread changes in gene expression, consistent with a global cellular response to metabolic stress. Among the differentially expressed genes, several have previously been implicated in stress adaptation, cell-cycle regulation, extracellular matrix remodeling, and metabolic homeostasis, including HSPA6, HSPA7, CDKN1A, CDC25A, MMP10, and HTRA3. Although the functional contribution of each individual gene was not investigated in the present study, their coordinated modulation is consistent with the profound metabolic and phenotypic alterations observed following CTP treatment.

Finally, this study demonstrates that the therapeutic value of CTP extends beyond direct tumor cytotoxicity by modulating the immune response within the cancerous stroma. In LPS-activated RAW 264.7 macrophages, the formulation markedly reduced nitric oxide (NO) production, as estimated by nitrite accumulation, together with the secretion of Interleukin-6 (IL-6) and Interleukin-8 (IL-8) in a concentration-dependent manner (Figure 8A–C). Given that the tumor microenvironment in both CRC and PDAC is inherently inflammatory and utilizes cytokines like IL-6 to perpetuate chemoresistance, immune evasion, and KRAS-mediated survival, the concomitant anti-inflammatory property of CTP represents a crucial clinical advantage.

An important limitation of the present study is that CTP was evaluated as a complete bioactivated formulation rather than as a purified compound or isolated metabolites. Therefore, the nominal concentrations used in vitro (mg/mL) refer to the whole tablet formulation, including plant material, excipients, and the enzymatic components required for glucosinolate bioactivation, rather than to the concentration of the bioactive sulfur-containing metabolites generated after hydrolysis. Consequently, the effective concentration of these metabolites, including CETP and related glucosinolate-derived products, are expected to be substantially lower than the nominal concentration of the formulation.

Importantly, significant biological effects were already observed at 1 mg/mL, indicating that the activity of CTP is not restricted to the highest concentration tested. Nevertheless, further studies are required to quantify the metabolites generated under physiological conditions, determine their pharmacokinetic and pharmacodynamic properties, and establish their effective concentrations in vivo.

Taken together, the data derived from this integrated experimental framework (Figure 2, Figure 3, Figure 4, Figure 5, Figure 6, Figure 7 and Figure 8) unequivocally demonstrates the potential of CTP as a versatile, naturally derived polypharmacological agent. By simultaneously destabilizing metabolic hubs (glycolysis/respiration), suppressing key oncoproteins (C-myc/PKM2), inducing transcriptomic cellular stress, and dampening the inflammatory component, this epithionitrile-rich formulation opens an innovative therapeutic window for the treatment of aggressive, traditionally refractory malignancies driven by oncogenic KRAS mutations.

Importantly, the novelty of the present study extends beyond confirming the anticancer activity of glucosinolate-derived compounds, which has been extensively documented. While previous studies have primarily focused on purified glucosinolates, isothiocyanates, or the pro-apoptotic activity of individual hydrolysis products, our work evaluates a chemically characterized bioactivated *Lepidium latifolium* formulation specifically designed to promote in situ epithionitrile generation. Moreover, by combining metabolic flux analyses, mitochondrial bioenergetics, transcriptomic profiling, modulation of KRAS-associated metabolic regulators, and anti-inflammatory activity, we provide a comprehensive mechanistic characterization that has not been reported for epithionitrile-enriched *L. latifolium* formulations. These findings broaden the current understanding of glucosinolate-derived metabolites and identify a multitarget strategy to exploit the metabolic vulnerabilities of KRAS-driven malignancies.

## 4. Materials and Methods

### 4.1. CTP Formulation

CTP is a proprietary bioactivated formulation developed from young leaves of *Lepidium latifolium* L. (*Brassicaceae*) by Soria Natural S.A. (Soria, Spain). The formulation consists of powdered young leaves (304.64 mg per tablet), dry leaf extract (205.70 mg per tablet), ascorbic acid, ferrous sulfate, and standardized amounts of sinigrin (30 mg/tablet), endogenous myrosinase activity (5 U/tablet), and epithiospecifier protein (ESP, 200 μg/tablet). The formulation was specifically designed to promote the in situ hydrolysis of sinigrin and the generation of epithionitriles, particularly 1-cyano-2,3-epithiopropane (CETP), after oral administration. The composition is protected by the Spanish patent “Detoxifying composition of alkenylglucosinolate” (ES2376716). Tablets were supplied by Soria Natural S.A. and freshly suspended in sterile culture medium immediately before each experiment.

For all in vitro experiments, CTP tablets were finely powdered and suspended in sterile culture medium at the desired concentrations (expressed as mg of whole CTP formulation per mL of culture medium), vortexed, and used immediately after preparation.

### 4.2. Sinigrin Quantification by HPLC-DAD

Sinigrin content was quantified by high-performance liquid chromatography coupled with diode-array detection (HPLC-DAD). Standard solutions of sinigrin were prepared in ultrapure water at concentrations of 25, 50, 75, and 100 ppm, filtered through 0.45 μm membrane filters, and injected in triplicate.

For sample preparation, 1.0 g of plant material was extracted with 50 mL of methanol under reflux at 70 °C for 30 min. The extract was concentrated to dryness and the residue was reconstituted in 100 mL of ultrapure water using several 30 mL portions assisted by ultrasound. An aliquot of 1 mL was diluted to 5 mL with ultrapure water and filtered through a 0.45 μm membrane filter before analysis.

Chromatographic separation was performed using a Zorbax Eclipse XDB-C18 column (4.6 × 250 mm, 5 μm; Agilent Technologies, Santa Clara, CA, USA). The mobile phase consisted of methanol (solvent A) and 30 mM ammonium acetate adjusted to pH 3.0 with formic acid (solvent B). The flow rate was 1.0 mL min^−1^, the injection volume was 20 μL, and detection was carried out at 233 nm using a diode-array detector.

### 4.3. Viability Assay

Two colon cancer cell lines (DLD-1 and SW480) and two pancreatic cancer cell lines (MIA PaCa-2 and PL45) with KRAS mutations were treated with increasing doses of CTP (1 mg/mL, 3 mg/mL, 5 mg/mL) for 24 h. Subsequently, the cells were trypsinized and stained with Trypan Blue for counting using a Neubauer chamber (Cultek, Madrid, Spain).

### 4.4. ATP Production Assay

This assay was performed following the Abcam protocol ab113849 (Waltham, MA, USA). Briefly, 15,000 cells of SW480, DLD-1, MIA PaCa-2, and PL45 were seeded in a 96-well plate and allowed to adhere overnight. The cells were then treated with 3 mg/mL of CTP for 24 h. At the end of the treatment, detergent was added to the wells to lyse the cells, and the corresponding substrate was added to measure luminescence.

### 4.5. Mitochondrial Membrane Potential Assay

This assay was conducted following Abcam protocol ab113850. In summary, 15,000 cells of SW480, DLD1, MIA PaCa-2, and PL45 were seeded in a 96-well plate and allowed to adhere overnight. The cells were then treated with 3 mg/mL of CTP for 24 h. FCCP (100 µM) was used as a positive control for mitochondrial membrane depolarization. Subsequently, the cells were stained with 20 µM of JC-1 for 10 min, and fluorescence was measured at 535/590 nm.

### 4.6. Western Blot

Three million cells of DLD-1, SW480, MIA PaCa-2, and PL45 were seeded and allowed to adhere overnight. The cells were then treated with 3 mg/mL of CTP for 24 h and collected using trypsin. Proteins were extracted using RIPA buffer supplemented with 1x protease and phosphatase inhibitors and quantified using the BCA method. Protein samples (20 µg protein/well) were resolved by SDS-PAGE (10%) and transferred to membranes, which were blocked for 1 h in 5% milk. The membranes were incubated overnight at 4 °C with primary antibodies against c-myc, PKM2, GLUT1, cyclin E1, and β-actin. After washing four times with TBS-T (0.1% Tween-20 in Tris-buffered saline, TBS), the membranes were incubated with peroxidase-conjugated anti-mouse and anti-rabbit secondary antibodies for 1 h at room temperature. The membranes were then washed four times with TBS-T and developed using ECL prime. Images were acquired using an Amersham Imager 600 (GE Healthcare, Chicago, IL, USA).

### 4.7. RT-qPCR Assays

Total RNA was isolated from SW480 and MIA PaCa-2 cells using the RNeasy Mini Kit (Qiagen, Hilden, Germany) following the manufacturer’s protocol. The quality and quantity of mRNA were assessed spectrophotometrically using a NanoDrop 2000 UV/Vis spectrophotometer (Thermo Fisher Scientific, Waltham, MA, USA). cDNA was synthesized from total mRNA using the Advantage RT-for-PCR Kit (Clontech Laboratories, Takara Bio USA, Inc., Palo Alto, CA, USA) following the manufacturer’s instructions. Target sequence PCR analyses were generated using the Advantage cDNA Kit (Clontech Laboratories) with the following PCR primers: GLUT1: (5′-TCATCAACCGCAACGAGGAG-3′ and 5′-CAAAGATGGC-CACGATGCTC-3′); LDHA: (5′-ACACGCCCATCCGAGCAGG-3′ and 5′-GCACAGGCACCAATTCCATAAAAC-3′).

PCR conditions: 95 °C for 1 min; 25 cycles of 95 °C for 30 s, 57 °C for 1 min, 72 °C for 2 min; 72 °C for 5 min. PCR products were separated on a 1% agarose gel containing GelRed™ nucleic acid stain (Biotium, Fremont, CA, USA) and visualized under UV light using a PhotoDyne imaging system (PhotoDyne Technologies, Los Angeles, CA, USA). cDNA and PCR products were generated using an MJ Mini Personal Thermal Cycler (Bio-Rad, Hercules, CA, USA).

### 4.8. OCR/ECAR Assay

In a 96-well plate, 2 × 10^4^ cells of MIA PaCa-2, PL45, SW480, and DLD-1 were seeded and allowed to adhere overnight. The following day, the cells were treated with CTP (3 mg/mL) for 6 h. Subsequently, oxygen consumption rate (OCR) and extracellular acidification rate (ECAR) were determined using the Seahorse XF Cell Mito Stress Test Kit (Agilent Technologies, CA, USA) following the manufacturer’s instructions. During the assay, four inhibitors of oxidative phosphorylation were serially injected as shown in the image. Oligomycin, a macrolide produced by Streptomyces bacteria, specifically blocks the proton channel in the FO portion of ATP synthase. The decrease in OCR following its injection provides information on mitochondrial ATP-linked respiration. Carbonyl cyanide-p-trifluoromethoxyphenylhydrazone (FCCP), a mobile ion carrier, acts as an uncoupling agent by disrupting ATP synthesis by transporting hydrogen ions across the membrane before they can be used to provide energy for oxidative phosphorylation. Measurements after its injection indicate the cell’s maximum respiration capacity. Rotenone and antimycin A, added together, inhibit complexes I and III of oxidative phosphorylation, providing information on the cell’s spare respiratory capacity, which is the difference between basal and maximum respiration.

### 4.9. Massive Sequencing Assays (RNA-Seq) in Human Pancreatic Cancer Cell Lines Treated with CTP

For the determination of genes whose expression is altered by CTP, we used the NGS massive sequencing technique.

Three million MIA PaCa-2 cells were seeded and allowed to adhere for 24 h. The following day, the cells were treated with ascorbic acid (5 mM) or CTP (5 mg/mL) for 6 and 12 h, adding them sequentially. After the treatments, the cells were collected using trypsin, and RNA was extracted using the Nucleospin Kit (Macherey-Nagel, Düren, Germany) following the indicated protocol. RNA was quantified by nanodrop, and dilutions of 50 ng/μL were prepared for subsequent submission to DIAGENODE S.A. for mRNA-seq analysis. Sequencing of the samples was performed on an Illumina NovaSeq 6000 instrument (San Diego, CA, USA), which produced paired-end reads of 50 bp using Control Software 1.7.0. The sequenced reads were quality-controlled using FastQC and trimmed with Trim_Galore. The clean reads were aligned to the reference genome using the STAR aligner version 2.7.9a [2], and RNA abundance was estimated using featureCounts from the Subread package version 2.0.3.

### 4.10. Inflammation Assay

RAW 264.7 mouse macrophages (CLS, Eppelheim, Germany, Ref. 400319) were seeded in 24-well plates at a density of 8 × 10^5^ cells per well and incubated at 37 °C in a humidified atmosphere containing 5% CO_2_ for 24 h. After this period, cells were treated with CTP (0.1–5 mg/mL). One hour after treatment, lipopolysaccharide (LPS) from *Escherichia coli* O127:B8 (Sigma-Aldrich, St. Louis, MO, USA, Ref. L5668) was added at a final concentration of 0.1 µg/mL.

After 24 h of incubation, culture supernatants were collected into microtubes and centrifuged at 1800 rpm for 10 min at 4 °C. The clarified supernatants were transferred to new microtubes and analyzed using the Griess Reagent System (Promega, Madison, WI, USA, Ref. G2930) to quantify nitrite accumulation. Samples were stored at −80 °C until further analysis.

Based on nitrite quantification results, selected samples were further analyzed for IL-6 production using a Murine IL-6 ELISA Kit (Diaclone, Besaçon Cedex, France, Ref. 860.020.192) and IL-8 production using a Murine IL-8 ELISA Kit (Fine Test, Wuhan, China, Ref. EM1595).

### 4.11. Statistical Analysis

Data are presented as mean ± standard deviation (SD) from at least three independent experiments unless otherwise indicated. Statistical analyses were performed using GraphPad Prism version 11.0.1 (GraphPad Software, San Diego, CA, USA). Comparisons between multiple groups were carried out using one-way analysis of variance (ANOVA) followed by Tukey’s post hoc multiple comparison test. Differences were considered statistically significant at *p* < 0.05.

For RNA-seq experiments, differential gene expression analysis was performed using the edgeR package (version 4.6.3) in R (version 4.5.x). Genes with adjusted false discovery rate (FDR) values < 0.05 were considered significantly differentially expressed.

## 5. Conclusions

The present study demonstrates that the glucosinolate-based formulation CTP, derived from *Lepidium latifolium* and enriched in bioactive sulfur-containing metabolites generated through controlled sinigrin bioactivation, exerts significant antitumor and anti-inflammatory activities in KRAS-mutant colorectal and pancreatic cancer models. CTP reduced cancer cell viability, impaired mitochondrial function and cellular bioenergetics, downregulated key metabolic and proliferative regulators, including C-myc, PKM2, GLUT1, and Cyclin E1, and induced extensive transcriptional reprogramming associated with oxidative stress, metabolic adaptation, and cell-cycle regulation.

In addition, CTP significantly suppressed the production of inflammatory mediators in activated macrophages, supporting a dual antitumor and anti-inflammatory mode of action. Collectively, these findings identify CTP as a chemically characterized, multitarget formulation capable of exploiting metabolic vulnerabilities associated with oncogenic KRAS signaling. Further studies are warranted to characterize the specific contribution of individual glucosinolate-derived metabolites, validate these effects in additional preclinical models, and explore the translational potential of this formulation as an adjuvant strategy for KRAS-driven malignancies.

## Figures and Tables

**Table 1:** Flavonoid composition of the CTP formulation determined by LC-Orbitrap high-resolution mass spectrometry. Individual flavonoids identified in the CTP formulation were quantified by LC-Orbitrap high-resolution mass spectrometry. Values are expressed as mean ± SD (mg/100 mL). Total flavonoid content corresponds to the sum of the quantified compounds.

Class	Compound	Content (mg/100 mL)
Flavan-3-ols	Catechin	75.31 ± 3.34
	Epicatechin	11.52 ± 0.18
	Epicatechin gallate	24.67 ± 1.24
Flavonol glycosides	Rutin	2571 ± 290
	Hyperoside + Isoquercitrin	1781 ± 68
	Reynoutrin + Guajaverin	185 ± 10
	Kaempferol-3-*O*-glucuronide	113.3 ± 4.2
Flavones/flavone glycosides	Luteolin-7-*O*-glucuronide	3994 ± 113
	Apigenin-7-*O*-glucoside	1340 ± 65
	Isorhoifolin	141.9 ± 4.8
	Rhoifolin	161.8 ± 5.5
	Apiin	77.3 ± 2.1
	Diosmin	1264 ± 131
	Luteolin	593.3 ± 60.7
	Apigenin	71.83 ± 6.63
Flavanones	Naringin	16.44 ± 1.51
	Hesperidin	22.2 ± 0.7
	Eriodictyol	55.8 ± 5.1
	Naringenin	166.2 ± 17.5
Flavonols	Quercetin	65.08 ± 5.74
	Kaempferol	66.12 ± 6.26
Total flavonoid content		12.8 mg/100 mL

**Table 2:** Top 19 differentially expressed genes identified by RNA-seq analysis in KRAS-mutant MIA PaCa-2 pancreatic cancer cells following CTP treatment. Differential gene expression analysis was performed using edgeR in MIA PaCa-2 cells treated with CTP (5 mg/mL, 24 h) compared with untreated control cells. Genes are ranked according to statistical significance (*p*-value) and include gene symbol, Ensembl transcript identifier, log2 fold change (logFC), *p*-value, and false discovery rate (FDR). Positive logFC values indicate upregulated genes in response to CTP treatment. Several transcripts correspond to stress-response proteins, metabolic regulators, cell-cycle-associated factors, and tumor-related signaling molecules, supporting extensive transcriptional reprogramming induced by CTP in KRAS-mutant pancreatic cancer cells. Some Ensembl identifiers could not be assigned to annotated gene names and may correspond to uncharacterized transcripts or pseudogenes.

Gene Name	Ensembl ID	logFC	*p* Value	FDR
MUC5AC	ENSG00000215182	14.175	1.46 × 10^−15^	2.696 × 10^−11^
HSPA6	ENSG00000173110	9.305	5.41 × 10^−15^	4.989 × 10^−11^
LPAR6	ENSG00000139679	8.435	8.90 × 10^−13^	5.223 × 10^−9^
ELAPOR1	ENSG00000116299	12.630	1.13 × 10^−12^	5.223 × 10^−9^
PCDHA3	ENSG00000255408	12.228	6.29 × 10^−12^	2.321 × 10^−8^
HSPA7	ENSG00000225217	11.915	2.40 × 10^−11^	7.373 × 10^−8^
SOWAHD	ENSG00000187808	11.717	5.51 × 10^−11^	1.462 × 10^−7^
MMP10	ENSG00000166670	11.591	9.49 × 10^−11^	2.189 × 10^−7^
NRCAM	ENSG00000091129	7.713	1.29 × 10^−10^	2.655 × 10^−7^
RADIL	ENSG00000157927	7.201	1.76 × 10^−10^	3.247 × 10^−7^
FNDC7	ENSG00000143107	11.281	3.54 × 10^−10^	5.043 × 10^−7^
ARC	ENSG00000198576	6.741	3.62 × 10^−10^	5.043 × 10^−7^
FAM83C	ENSG00000125998	6.974	3.83 × 10^−10^	5.043 × 10^−7^
FNDC11	ENSG00000125531	11.214	4.69 × 10^−10^	5.776 × 10^−7^
HMOX1	ENSG00000100292	6.538	5.72 × 10^−10^	6.598 × 10^−7^
IQCA1L	ENSG00000278685	11.114	7.05 × 10^−10^	7.651 × 10^−7^
ABCA9	ENSG00000154258	7.985	7.60 × 10^−10^	7.793 × 10^−7^
SLC5A10	ENSG00000154025	11.077	8.24 × 10^−10^	8.006 × 10^−7^
RYR1	ENSG00000196218	7.835	1.42 × 10^−09^	1.312 × 10^−6^

## Data Availability

The data presented in this study are available on request from the corresponding author.

## Abbreviations

The following abbreviations are used in this manuscript:

CRC	Colorectal cancer
CTP	*Lepidium latifolium*-derived formulation
CETP	1-cyano-2,3-epithiopropane
ECAR	Extracellular acidification rate
ESP	Epithiospecifier protein
FDR	False discovery rate
GLUT1	Glucose transporter 1
IL-6	Interleukin-6
IL-8	Interleukin-8
KRAS	Kirsten rat sarcoma viral oncogene homolog
LPS	Lipopolysaccharide
NO	Nitric oxide
OCR	Oxygen consumption rate
PDAC	Pancreatic ductal adenocarcinoma
PKM2	Pyruvate kinase M2
ROS	Reactive oxygen species
